# One-step synthesis of nitrogen-grafted copper-gallic acid for enhanced methylene blue removal

**DOI:** 10.1038/s41598-021-91484-w

**Published:** 2021-06-08

**Authors:** Shella Permatasari Santoso, Vania Bundjaja, Artik Elisa Angkawijaya, Chintya Gunarto, Alchris Woo Go, Maria Yuliana, Phuong Lan Tran-Nguyen, Chang-Wei Hsieh, Yi-Hsu Ju

**Affiliations:** 1 Department of Chemical Engineering, Widya Mandala Catholic University Surabaya, Kalijudan 37, Surabaya, 60114 Indonesia; 2 Department of Chemical Engineering, National Taiwan University of Science and Technology, No. 43, Sec. 4, Keelung Rd., Taipei, 10607 Taiwan; 3 Graduate Institute of Applied Science and Technology, National Taiwan University of Science and Technology, No. 43, Sec. 4, Keelung Rd., Taipei, 10607 Taiwan; 4 Department of Mechanical Engineering, Can Tho University, 3-2 Street, Can Tho City, Vietnam; 5 Department of Food Science and Biotechnology, National Chung Hsing University, No. 145 Xingda Road, South District, Taichung City, 40227 Taiwan; 6 Taiwan Building Technology Center, National Taiwan University of Science and Technology, No. 43, Sec. 4, Keelung Rd., Taipei City, 10607 Taiwan

**Keywords:** Materials science, Environmental impact

## Abstract

Nitrogen-grafting through the addition of glycine (Gly) was performed on a metal- phenolic network (MPN) of copper (Cu^2+^) and gallic acid (GA) to increase its adsorption capacity. Herein, we reported a one-step synthesis method of MPN, which was developed according to the metal–ligand complexation principle. The nitrogen grafted CuGA (N_*g*_-CuGA) MPN was obtained by reacting Cu^2+^, GA, and Gly in an aqueous solution at a molar ratio of 1:1:1 and a pH of 8. Several physicochemical measurements, such as Fourier transform infrared (FTIR) spectroscopy, scanning electron microscopy (SEM), N_2_ sorption, X-ray diffraction (XRD), and thermal gravimetry analysis (TGA), were done on N_*g*_-CuGA to elucidate its characteristics. The analysis revealed that the N_*g*_-CuGA has non-uniform spherical shaped morphology with a pore volume of 0.56 cc/g, a pore size of 23.25 nm, and thermal stability up to 205 °C. The applicational potential of the N_*g*_-CuGA was determined based on its adsorption capacity against methylene blue (MB). The N_*g*_-CuGA was able to adsorb 190.81 mg MB per g adsorbent at a pH of 6 and temperature of 30 °C, which is 1.53 times higher than the non-grafted CuGA. Detailed assessment of N_*g*_-CuGA adsorption properties revealed their pH- and temperature-dependent nature. The adsorption capacity and affinity were found to decrease at a higher temperature, demonstrating the exothermic adsorption behavior.

## Introduction

A recent trend in metal–ligand coordination research expands to the use of plant polyphenols as metal ions linkers in producing metal-phenolic networks (MPNs)^1^. In principle, MPNs are formed via coordination between metal ions and phenolic acids. This kind of coordination can be observed in biological functions; for example, the formation of the Mg^2+^/porphyrin MPN during plant photosynthesis^2^. In application, MPNs are commonly adopted as conformal coats of various substrates to promote their functionality, pH responsiveness, biocompatibility, and bioavailability^3–6^. MPNs also have gained considerable attention due to their tunable lipo/hydrophilic properties^7^. For instance, MPN prepared from a combination of Fe^3+^-tannic acid (FTA) is reported to enhance the hydrophobicity of Zeolitic Imidazolate Framework-8 (ZIF-8). Impregnation of FTA/ZIF-8 onto the fibrous substrate (i.e., kapok fiber core) creates a superhydrophobic sorbent that can be used to treat oily water^8^. Due to their properties, the usage of MPNs has been extended into various applications such as water treatment, pharmacological, imaging, sensor, biofouling agent, separation application, etc^3,9–13^. The use of MPN as an adsorbent has become one of its widely explored emerging applications. A study by Rahim et al. (2020) reported the preparation of an adsorbent from the combination of TA/Zn(IV) MPN for capturing heavy metal contaminants in wastewater^14^. Wang et al. (2019) assembled Ni-, Mg-, and Co-gallate with a tunable aperture for the adsorption and separation of acetylene from ethylene^15^. Within the range of available phenolic linkers, TA, gallic acid (GA), and polydopamine (PDA) are the widely used linker for MPN synthesis^16–19^. However, as TA and PDA overdose is postulated to cause adverse effects on human health and the environment^20,21^; therefore, GA was chosen in this work. Besides this toxicity issue, recently, our group reported the potential usage of CuGA as an adsorbent for dye removal^22^; thus, functionalization of this particular MPN is expected to improve their adsorptivity.

**Scheme 1:**
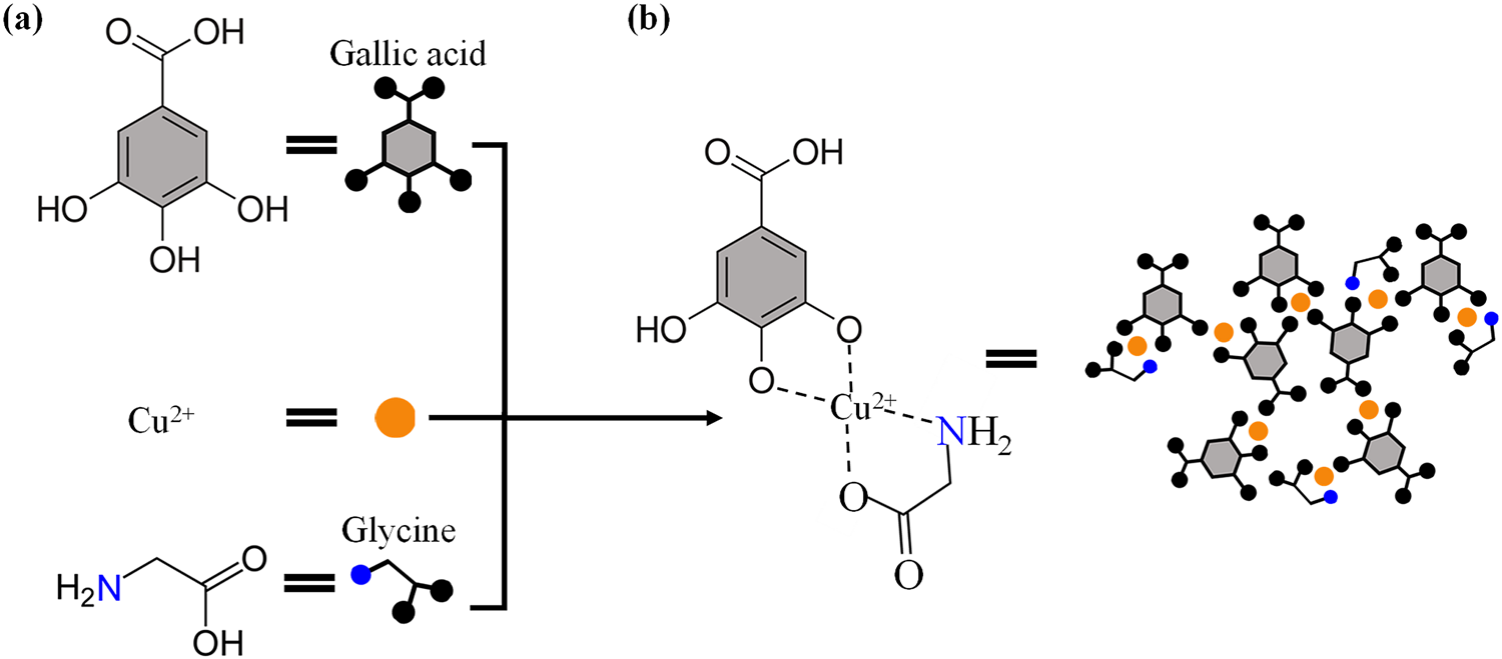
Illustration of the N_*g*_-CuGA assembly process. (**a**) Molecular structure of the individual ligands. (**b**) Molecular structure of the possible coordination between gallic acid (GA), glycine (Gly), and copper (II) ion (Cu^2+^).

Nitrogen-grafting (N_g_) is one of the surface functionalization techniques and had been acknowledged to promote the adsorption capacity of adsorbents^23–26^. Due to this advantage, various N_g_ methods have been extensively developed via plasma, pyrolysis, radiation, hydrothermal, or basic hydrolysis reaction^27–31^. This study utilizes the possible binding interaction of copper (Cu^2+^), GA, and amino acids through the metal–ligand complexation principle as the foundation for the N_*g*_-CuGA synthesis^32–34^. To the best of our knowledge, there is no N_g_-MPNs have been synthesized in similar methods nor applied for a similar purpose. Herein, we reported the modification of CuGA by the addition of glycine (Gly) as the modifying agent to provide a nitrogen functional group on the prepared MPN (Scheme 1). The synthesized N_*g*_-CuGA were examined using X-ray diffraction (XRD), Fourier transform infrared (FTIR) spectroscopy, scanning electron microscopy (SEM), thermal gravimetry analysis (TGA), and N_2_ sorption analysis to elucidate its physicochemical properties. The adsorption performance of N_*g*_-CuGA for methylene blue (MB) removal in an aqueous system was investigated.

## Materials and methods

### Materials

The chemicals used were of analytical grade and were immediately used without further purification. GA (C_7_H_6_O_5_, 0.98 purity) was obtained from the Tokyo Chemical Industry (Tokyo, Japan). Gly (C_2_H_5_NO_2_, 0.99 purity) was obtained from Sigma (Steinheim, Germany). Copper chloride dihydrate (CuCl_2_·2H_2_O, 0.99 purity) was purchased from Kanto Chemical Co., Inc. (Japan). Sodium hydroxide (NaOH, 0.96 purity) was obtained from Yakuri Pure Chemical (Japan). Sodium chloride (NaCl, 0.995 purity) and sodium carbonate (Na_2_CO_3_, 0.998 purity) were obtained from Showa Chemical and Nacalai Tesque (Japan), respectively. MB (C_16_H_18_N_3_SCl·3H_2_O, 0.95 purity) was acquired from Sigma Aldrich (St. Louis, MO). Ethanol (C_2_H_6_O, 0.95 purity) was purchased from Echo Chemical (Taiwan). The solutions used for the experiments were prepared in ultra-pure water with a resistance of 18.3 MΩ cm^-1^, which was produced from purification by a NANO Ultrapure water system.

### Synthesis of the N_g_-CuGA MPN

10 mL of aqueous CuCl_2_·2H_2_O (5 mmol) solution was slowly added to the 10 mL aqueous mixture of GA (5 mmol) and Gly (5 mmol). To the mixture, 0.1 M NaOH was added until it reaches pH 8. The reaction was done for 4 h, under constant stirring. The synthesized N_*g*_-CuGA was then collected by centrifugation, washed several times with ethanol to remove any unreacted reactants, and subjected to drying at 50 °C under vacuum.

### Characterization of the complexes

The FTIR spectra were recorded using a Bio-Rad FTS 3500 FT-IR spectrophotometer in KBr pellets and a wavenumber range of 4000 to 400 cm^-1^. The surface topography imaging was done using a JEOL JSM-6500F Scanning Electron Microscope. XRD patterns were recorded using an X-ray Diffractometer Bruker D2 Phaser with Cu-Kα radiation (λ = 1.54184 Å) at 30 kV and 10 mA. The isotherms of N_2_ adsorption–desorption were measured at 77 K using a BELSORP-max analyzer; samples were degassed for 12 h at 423 K before analysis. The specific surface area was calculated from the adsorption branch using the Brunauer–Emmett–Teller (BET) model. TGA analysis was carried out using a Perkin Elmer Diamond TG/DTA in the temperature range of 30–600 °C and a heating rate of 10 °C/min under N_2_ gas flow. Elemental analysis of the complex was carried out using a Thermo Flash 2000 CHNS/O Analyzers, while the copper metal content was analyzed using a JY2000-2 Inductively Coupled Plasma Atomic Emission Spectroscopy. The absence of chloride in the N_*g*_-CuGA was confirmed using a Dionex ICS-1000 with NaCl as a standard. pH_pzc_ determination was performed using a procedure described by Angkawijaya et al.^35^, briefly: a series of 0.1 M NaCl solutions with adjusted pH (2 to 10) were prepared in capped vials at a volume of 5 mL. The 15 mg of sample was added to each vial and allowed to contact for 48 h in a shaking incubator operated at 200 rpm and constant temperature of 30˚C. The measures of final pH were made by using a pH meter (Denver Instrument UB-10). The pH_final_ was plotted against the pH_initial_, the intersection of the experimental curve with the pH_initial_ = pH_final_ linear plot was identified as pH_pzc_.

### Adsorption study

#### Effect of pH

The investigation on the effect of pH on MB adsorption was carried out in a series of micro-test tubes containing 2 mL of pH-adjusted MB solution at the initial concentration of 500 mg/L. 20 mg adsorbents were added to these working solutions and were incubated at 30 °C with constant shaking at 200 rpm. After 24 h, the adsorbents were separated from the solutions by centrifugation at 15,000 rpm for 10 min. The concentration of remaining MB in the solution was determined using Spectrophotometer UV–Vis (Shimadzu UV-2600) at λ_max_ = 664 nm. The adsorption capacity, which is the amount of MB adsorbed per g of adsorbent, *Q*_e_ (mg/g), was calculated using Eq. (1).

1 $$Q_{e} = \left( {\frac{{C_{0} - C_{{\text{e}}} }}{m}} \right) \times V$$

where *C*_0_ and *C*_e_ (mg/L) are the initial and equilibirum concentrations of MB, respectively. *V* (L) is the total volume of the investigated system, and *m* (g) is the mass of adsorbent.

#### Effect of adsorbent dosing and salinity

The N_*g*_-CuGA adsorbent at different dosage ranging from 0.2 to 1.1 mg/L were introduced into the working solutions of MB at initial concentration of 70 ppm. After 24 h incubation at 30 °C, the residual concentration of MB was measured by UV–Vis spectrophotometer. The amount of MB that can be removed at the different adsorbent dosing was calculated according to Eq. (2).

2 $${\text{\% Removal}} = \left( {\frac{{C_{0} - C_{f} }}{{C_{0} }}} \right) \times 100$$

where *C*_*f*_ is the residual concentration of MB in the bulk solution (mg/L).

The effect of salinity was investigated by preparing MB solution in the presence of different salts. The salt-containing solutions were prepared by dissolving 2000 mg of MB in 1 L of water containing either 20 mg Na_2_CO_3_, 300 mg NaCl, or a combination of both salt (20 mg Na_2_CO_3_ and 300 mg NaCl). Subsequently, to the 20 mL of these solutions, 20 mg of the adsorbent was added. The %adsorption efficiency was then calculated by comparing the adsorption capacity of N_*g*_-CuGA for MB removal in the salt-containing system (*Q*_salt_) to the control (no-salt system, *Q*_*control*_), according to Eq. (3).

3 $$\% {\text{Adsorption efficiency}} = \frac{{Q_{{{\text{salt}}}} }}{{Q_{{{\text{control}}}} }} \times 100$$

#### Adsorption isotherm study

A series of 2 mL MB solutions was prepared in various concentrations without any pH adjustment. Subsequently, 20 mg of adsorbent was added to these solutions and shaken vigorously. The adsorption was conducted at three different temperatures (30, 40, and 50 °C). After 24 h, the concentration of residual MB at the end of the adsorption was measured using a UV–Vis spectrophotometer (Shimadzu UV 2600)^22^. The adsorption data were plotted as $Q_{e}$ versus $C_{e}$ and were fitted using two-parameter isotherm models (Langmuir, Freundlich, and Temkin) and three-parameter models (Sips and Redlich–Peterson).

The Langmuir model, which accounts for monolayer surface coverage over a homogenous adsorbent surface, is mathematically expressed as Eq. (4):

4 $$Q_{e} = \frac{{Q_{L} \cdot K_{L} \cdot C_{e} }}{{1 + K_{L} \cdot C_{e} }}$$

where $Q_{e}$ is the quantity of MB adsorbed at equilibrium (mg/g), $C_{e}$ is the concentration of residual MB at equilibrium (mg/L), $K_{L}$ is the Langmuir affinity constant (L/mg), and $Q_{L}$ is the maximum adsorption capacity (mg/g)^36^.

The Freundlich model which able to describe the multilayer adsorption with the interaction between adsorbed molecules mathematically expressed as Eq. (5):

5 $$Q_{e} = K_{F} \cdot C_{e}^{{\frac{1}{{n_{F} }}}}$$

where $K_{F}$ is the Freundlich adsorption capacity in units of (mg/g)(mg/L)$^{n_{F}}$ and $1/n_{F}$ is a dimensionless parameter characterizing the heterogeneity^37^.

The Temkin model presumes a linear rather than a logarithmic decrease of adsorption heat as an increase of surface coverage by ignoring the lowest and highest extreme of the concentration. The equation given in Eq. (6) characterizes the uniform binding energy distribution.

6 $$Q_{e} = B \cdot \ln \left( {A_{T} \cdot C_{e} } \right)$$

7 $$B = \frac{R \cdot T}{b}$$

where $R$ is the universal gas constant (8.314 J/mol K), $T$ is the temperature (K), $b$ is the Temkin isotherm constant, and $A_{T}$ is equilibrium binding constant (L/mg)^38,39^.

The Sips and Redlich–Peterson models are often employed as confirmatory of Langmuir and Freundlich models. Sips model is the modified form of the Freundlich equation that obeys the continuous increase of capacity as an increase of concentration but has a finite limit at the sufficient high concentration^40^. The Redlich–Peterson is a versatile model applied to both homogenous and heterogeneous systems^39,40^. The equations are mathematically given as Eqs. (8) and  (9) for Sips and Redlich–Peterson, respectively.

8 $$Q_{e} = \frac{{Q_{S} \cdot a_{s} \cdot C_{e}^{{s_{p} }} }}{{1 + a_{s} \cdot C_{e}^{{s_{p} }} }}$$

9 $$Q_{e} = \frac{{K_{RP} \cdot C_{e} }}{{1 + a_{RP} \cdot C_{e}^{\beta } }}$$

In the Sips model, $Q_{S}$ is the Sips maximum adsorption capacity (mg/g), $a_{s}$ is the Sips equilibrium constant related to the adsorption affinity (L/mg), and $s_{p}$ is the Sips model exponent that expresses the heterogeneity of the adsorbent. Sips model reduces to Langmuir model as the $s_{p}$ = 1, and reduces to Freundlich when either $C_{e}$ or $a_{s}$ close to 0. In the Redlich–Peterson model, $K_{RP}$ (L/g) and $a_{RP}$ (L/mg) are the Redlich–Peterson constants for calculating the Redlich–Peterson adsorption capacity ($Q_{RP} = K_{RP} /a_{RP}$). Parameter $\beta$ is the Redlich–Peterson exponent. The equation reduces to Langmuir model as $\beta$ = 1 and reduces to Freundlich model as $a_{RP} \cdot C_{e}^{\beta }$ > 1^35,41^.

#### Adsorption thermodynamic

The thermodynamic parameters of the adsorption system such as Gibb’s free energy (Δ*G*, kJ), enthalpy (Δ*H*, kJ), and entropy (Δ*S*, J/K) were determined by the following van’t Hoff equation:

10 $$\Delta G = \Delta H - T \cdot \Delta S$$

11 $$\Delta G = - RT\ln K_{C}$$

12 $$\ln K_{C} = - \frac{\Delta H}{{RT}} + \frac{\Delta S}{R}$$

where Δ*G* can be calculated by using Eq. (11) and $K_{C}$ (dimensionless) value could be obtained by multiplying *K*_L_ (L/mg) by the molecular weight of the adsorbate (g/mol) by 1000 (conversion from gram to mg) and then by 55.5 (the number of moles of pure water per liter)^42^. The value of Δ*H* and Δ*S* are obtained as the slope and intercept of ln *K*_C_ versus 1/*T* plot Eq. (12)^43^.

#### Adsorption kinetics study

A series of 2 mL MB solutions was prepared at a specific initial concentration of 500 ppm and 2000 ppm. To these working solutions, 20 mg of the adsorbent was added. At certain incubation time (*t*, hour), the residual concentration of the MB (*C*_*t*_, mg/L) was measured, and the amount of MB adsorbed at specific *t* (*Q*_t_, mg/g) was calculated. The Pseudo first order Eq. (13) and Pseudo second order Eq. (14) equation were used to fit the *Q*_t_ versus *t* plot and predict the adsorption rate of MB removal.

13 $$Q_{t} = Q_{1} \cdot \left( {1 - e^{{ - k_{1} t}} } \right)$$

14 $$Q_{t} = Q_{2} \cdot \left( {\frac{{Q_{2} k_{2} t}}{{1 + Q_{2} k_{2} t}}} \right)$$

where *Q*_1_ and *Q*_2_ are the adsorption capacity as predicted by the model (mg/g); *k*_1_ (1/h) and *k*_2_ (g/mg h) are the adsorption rate for each model.

#### Reusability study

Twenty mg of the freshly synthesized N_*g*_-CuGA was introduced into 2 mL of 2000 ppm MB solution. The adsorption was allowed to proceed for 24 h at 30 °C. The MB containing N_*g*_-CuGA was then objected for the reusability study by first regenerating the adsorbent. The regeneration was done by immersing the post-adsorption adsorbent in 0.5 mL ethanol. Then, the adsorbent was dried in a 50 °C oven overnight before used for another adsorption cycle.

## Results and discussion

### Characterization of the N_g_-CuGA

**Figure 1:**
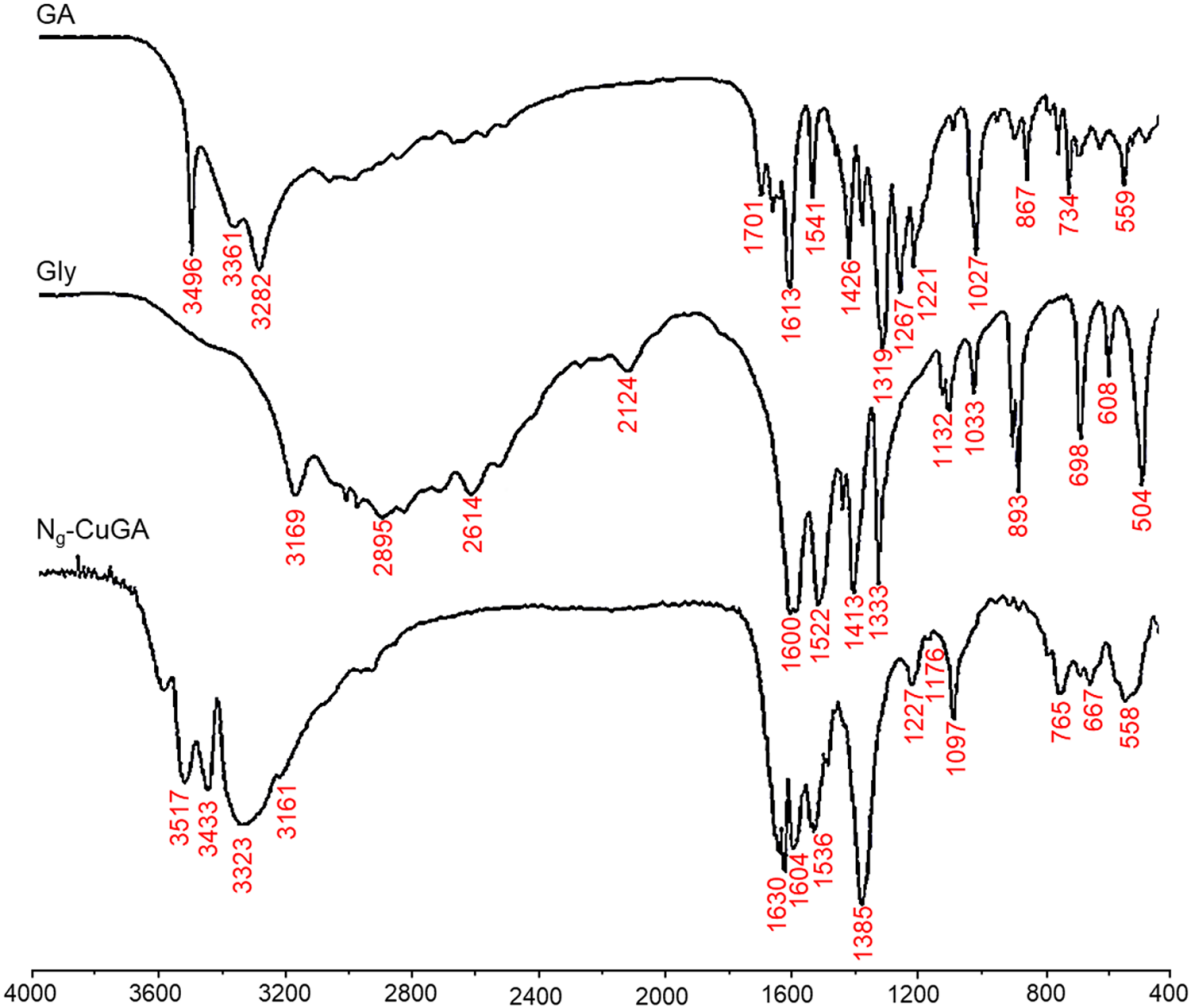
FTIR spectra of N_*g*_-CuGA MPN and the parent ligands.

N_*g*_-CuGA complexes were obtained by reacting equimolar amounts of GA, Gly, and Cu^2+^ in an aqueous solution, at pH 8 and room temperature. The elemental analysis and ICP results revealed that the brown-colored N_*g*_-CuGA has the compositions (%) of C, 30.044; H, 1.923; N, 1.104; O, 35.302; and Cu, 31.628. Several physicochemical measurements were also conducted to elucidate the characteristics of the N_*g*_-CuGA. The functional group bands of N_*g*_-CuGA were recorded using the FTIR spectrophotometer, and the result is depicted in Fig. 1. The FTIR spectra of the parent ligands (i.e., GA and Gly) were also given for comparison.

As presented in Table 1, the characteristic peaks of parent ligands (Gly and GA) can be observed in the N_*g*_-CuGA FTIR spectra. The incorporation of Gly to the CuGA was confirmed by the occurrence of characteristic peaks of Gly (N–H group vibration) at wavenumber 3161 cm^-1^ in N_*g*_-CuGA spectra^44^. The shifting of the OH groups was observed between wavenumber 3282–3496 cm^-1^. These indicate the involvement of the NH groups of Gly and OH groups of GA during N_*g*_-CuGA formation^45^.

**Table 1:** Selected FTIR spectrums of the parent ligands (GA, Gly) and N_*g*_-CuGA.

IR spectra (cm^-1^)			Assignment
Gly	GA	N_*g*_-CuGA	
1033	1027	1097	*v*(C–O) or *v*(C–N)
1333	1319	1385	*v*(OH)—bending
1413	1426	1424	*v*(C–O) or *v*(C–N)
–	1541	1540	*v*(C = C)
3169	–	3161	*v*(NH)
–	3282	3329	*v*(OH)—carboxyl
–	3496	3421	*v*(OH)—hydroxyl

**Figure 2:**
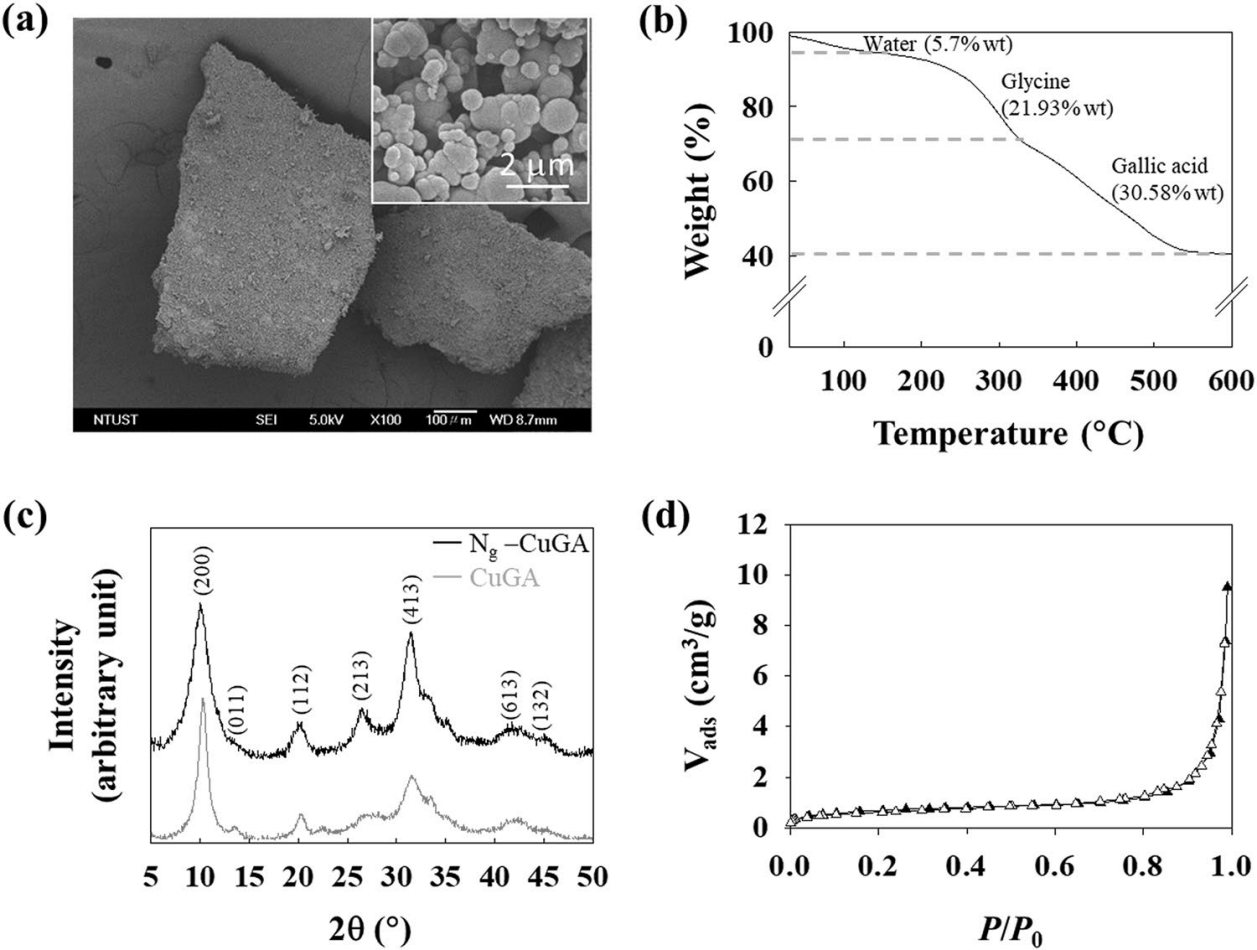
(**a**) FE-SEM of N_*g*_-CuGA with bulky shape, inset figure show spherical-like particles at 5000 × magnification. (**b**) TGA, (**c**) powder X-ray diffraction, and (**d**) nitrogen adsorption–desorption isotherm of N_*g*_-CuGA.

Based on the SEM image (Fig. 2a), it can be observed that the N_*g*_-CuGA is globular-shaped and has a non-uniform size ranging between 15 and 1500 nm in diameter. TGA was employed to determine the thermal stability of N_*g*_-CuGA. As shown in Fig. 2b, three stages of thermal decomposition were observed. The first stage thermal decomposition occurred between 30 and 151 °C, which corresponded to the loss of water (5.72 wt.%) and was followed by the decomposition of Gly (21.93 wt.%) at a temperature range of 205–321 °C. The last stage of decomposition is due to the loss of the GA molecule, which leaving CuO as the final residue (41.77 wt.%).

The crystalline structure and the phase characterization of N_*g*_-CuGA were obtained using powder XRD (Fig. 2c). The PXRD spectra of N_*g*_-CuGA showed a close resemblance to the diffraction spectra of unmodified CuGA^22^, specifically at 2θ = 10.1, 13.2, 20.1, 28.0, 31.1, and 42.8°. N_2_ adsorption/desorption analysis was performed to determine the surface area and pore properties of the N_*g*_-CuGA. As shown in Fig. 2d, a typical type IV isotherm curve with a hysteresis loop was observed. Based on the BET calculation, the N_*g*_-CuGA has a surface area of 2.00 m^2^/g, with pore volume and average pore diameter of 0.6 cc/g and 23.3 nm, respectively. Compared to the non-grafted CuGA (surface area of 198 m^2^/g, pore volume of 0.4 cc/g, and average pore diameter of 8.6 nm)^22^, the N_*g*_-CuGA possess a larger pore size. This phenomenon might be occurred due to the incorporation of small-grafting molecules (i.e., Gly), which may strain the internal pores of CuGA, and eventually caused a significant reduction in the surface area of N_*g*_-CuGA^24,46^. Although incorporating Gly is shown to trigger pore blockage, it is postulated that the nitrogen groups from the grafted molecules may act as the additional adsorption sites^24^, which can promote the adsorption capacity of CuGA.

### Adsorption study

#### Effect of pH

**Figure 3:**
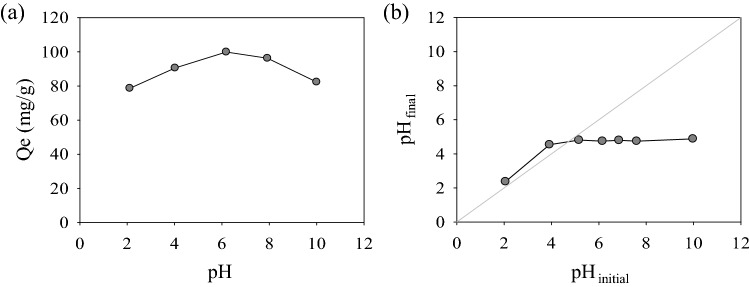
(**a**) Effect of initial pH on the adsorption of MB and (**b**) pH_pzc_ determination plot of N_*g*_-CuGA.

The pH of the solution is acknowledged as one of the important factors that affect the adsorption performance of the adsorbent^47^. As presented in Fig. 3a, optimum adsorption capacity (Qe 100.44 mg/g) of N_*g*_-CuGA can be achieved at pH 6. This adsorption capacity is gradually decreased as the pH of the solution transition to either lower or higher pH. The decline of adsorption capacity at pH > 6 might be attributed to the presence of numerous OH-oxyanions in the solution^48^. These negatively charged oxyanions may interact with the MB molecules, thus inhibit the electrostatic attraction between the N_*g*_-CuGA and MB. On the other hand, at pH lower than 6, the electrostatic repulsion between the N_*g*_-CuGA and MB might occur since both molecules are positively charged.

**Figure 4:**
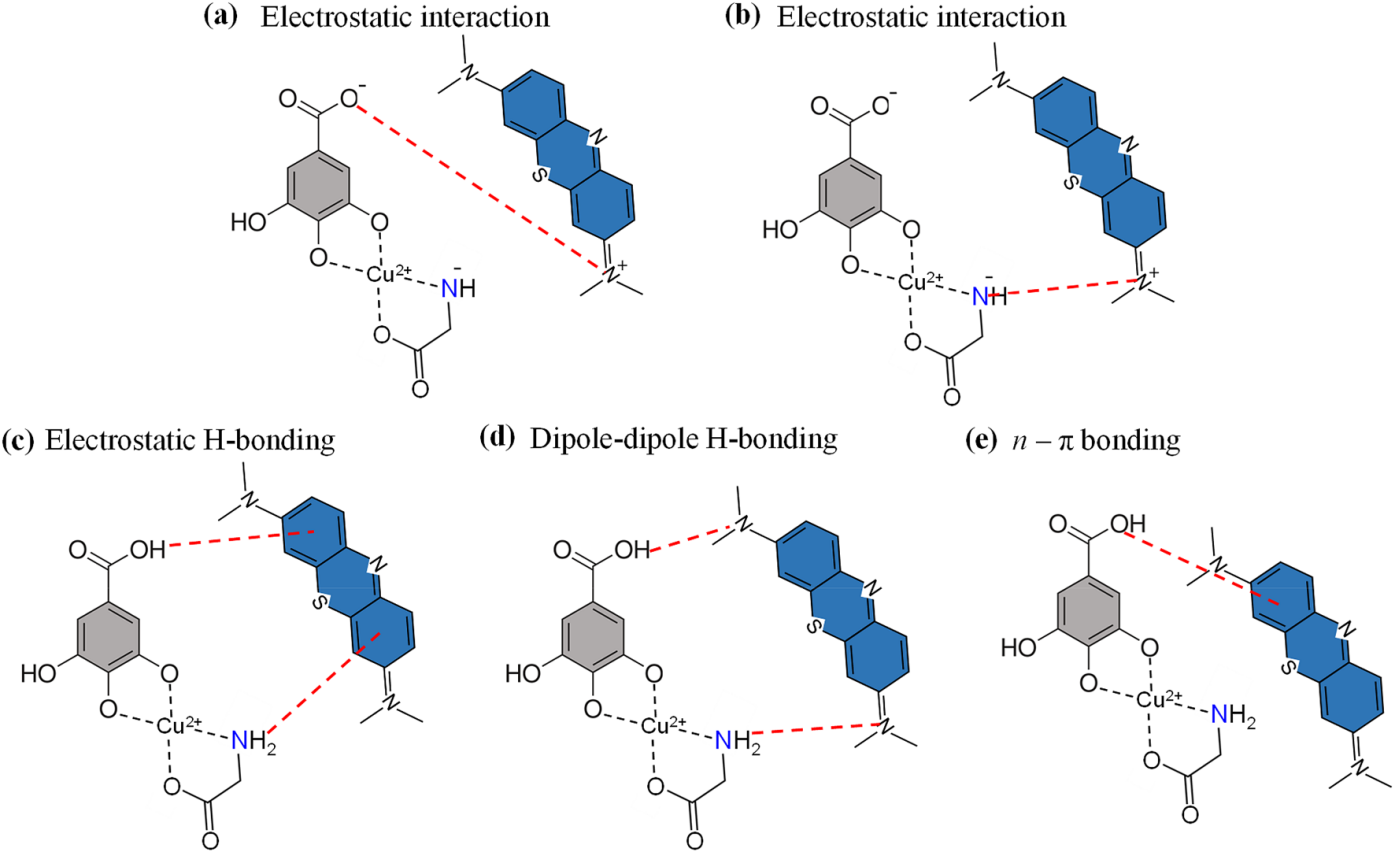
Possible interactions that induce adsorption of MB by N_*g*_-CuGA.

The highest MB adsorption by N_*g*_-CuGA occurred at pH 6 due to the contrasting charges of the adsorbent and adsorbate in this environment, which consequently induce their electrostatic interaction (Fig. 4a,b)^49^. The negative surface charge of N_*g*_-CuGA was confirmed through the pH_pzc_ value; wherein, it was found that the pH_pzc_ of N_*g*_-CuGA is 4.73 (Fig. 3b). When the pH of a solution is higher than pH_pzc_ (i.e., pH of 6), the surface charge of N_*g*_-CuGA tends to be negative due to the release of proton (H^+^) ions from the functional groups–COOH, –OH, and –NH_2_. While most N_*g*_-CuGA particles have a negative surface charge at pH of 6, some of their functional groups may remain protonated. These protonated N_*g*_-CuGA postulated to contribute to MB adsorption through several possible interactions such as electrostatic H-bonding, dipole–dipole H-bonding, and *n* *−* *π* bonding (Fig. 4c–e)^50,51^.

#### Adsorption isotherm

**Figure 5:**
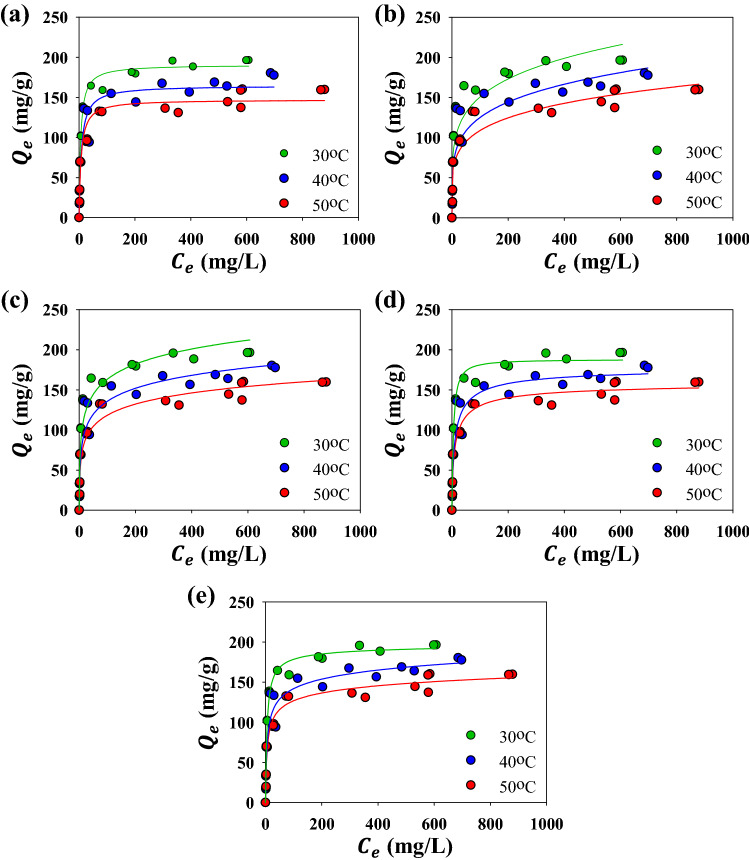
Adsorption isotherm of MB onto N_*g*_-CuGA. The solid lines represent the fits of Langmuir (**a**), Freundlich (**b**), Temkin (**c**), Sips (**d**), and Redlich–Peterson (**e**) isotherm models; symbols represent experimental data.

Adsorption isotherm study was carried out to predict the interaction mechanisms of MB and N_*g*_-CuGA at a constant temperature. The experiment was conducted at an initial solution pH of 6 since the adsorption proceeds optimally at this pH (see Fig. 3) and to eliminate the possibility of unexpected ion interaction or adsorption sites competition. The prediction of the interaction mechanisms can be made by investigating the isotherm curve shape and modeling the isotherm data^39^. Figure 5 shows the isotherm curves shape of MB adsorption by N_*g*_-CuGA at different temperatures. The adsorption data points line up to form a curve with a vertical orientation at low *C*_e,_ which can be classified as an H-type isotherm, according to the classification by Giles^52^. This type of isotherm is commonly observed when the adsorption process happened due to the electrostatic forces between the adsorbent and adsorbate. Based on the subclass classification of the isotherm curve, the adsorption of MB by N_g_-CuGA is classified as subclass 2. Subclass 2 commonly represents the high-affinity interaction between the solute adsorbate molecules with the solvent, but a low-affinity interaction between the adsorbed-adsorbate molecules and adsorbate molecules in bulk solution. This behavior is also indicated by the formation of a long plateau that signifies the adsorbent saturation^53^. The adsorption capacity based on experimental value (*Q*_exp_) is given in Table 2; it can be noted that the *Q*_exp_ is decreased as the adsorption temperature increases. This decrease is postulated due to the rise of MB solubility at higher temperatures which consequently hinder its affinity to the adsorbent^54^.

**Table 2:** Experimental maximum capacity and calculated isotherm parameters for the adsorption of MB onto N_*g*_-CuGA.

	30 °C	40 °C	50 °C
*Q*_exp_ (mg g^-1^)	196.480	180.458	160.238
**Langmuir**			
*Q*_L_ (mg g^-1^)	190.805 ± 4.214	164.854 ± 6.523	147.323 ± 4.688
*K*_L_ (L mg^-1^)	0.178 ± 0.019	0.123 ± 0.029	0.137 ± 0.028
R^2^	0.978	0.927	0.940
**Freundlich**			
*K*_F_ (L g^-1^)	59.884 ± 8.129	48.270 ± 6.909	44.111 ± 5.882
*n*_F_	4.992 ± 0.646	4.839 ± 0.603	5.102 ± 0.595
R^2^	0.862	0.885	0.903
**Temkin**			
*B* (J mol^-1^)	27.195 ± 1.92	23.329 ± 1.633	20.048 ± 1.315
*A*_T_ (L mg^-1^)	4.047 ± 0.35	3.374 ± 0.374	3.666 ± 0.379
R^2^	0.934	0.936	0.942
**Sips**			
*Q*_S_ (mg g^-1^)	188.172 ± 4.763	179.985 ± 13.150	160.498 ± 10.104
*a*_s_ (L g^-1^)	6.307 ± 1.127	5.479 ± 1.394	5.317 ± 1.144
*s*_p_	1.105 ± 0.119	0.692 ± 0.136	0.679 ± 0.125
R^2^	0.979	0.940	0.954
**Redlich–Peterson**			
*K*_RP_ (L g^-1^)	36.099 ± 5.234	33.966 ± 11.390	29.060 ± 7.525
*a*_RP_ (L mg^-1^)	0.208 ± 0.056	0.342 ± 0.171	0.311 ± 0.125
*β*	0.983 ± 0.025	0.913 ± 0.034	0.924 ± 0.030
R^2^	0.978	0.946	0.958

The adsorption data modeling was done using several adsorption isotherm models (i.e., Langmuir, Freundlich, Temkin, Sips, and Redlich–Peterson); the corresponding fitting model can be seen as the solid lines in Fig. 5. The two-parameters Langmuir and Freundlich model commonly employed to specify the adsorption behavior of the system, while the use of Temkin model is necessary to predict the heat transfer direction of the adsorption system. The value of parameters resulted from the experimental data fitting was listed in Table 2. The Langmuir model fit the MB adsorption by N_*g*_-CuGA better than the Freundlich model; this was indicated from the higher linear correlation coefficients (R^2^) of the Langmuir model.

The fitting result is consistent with the previous classification by Giles^52^, where most adsorption systems with an H type and subclass 2 curves are well convergence with the Langmuir model. The fitting using the Temkin model also shown a satisfactory R^2^ value of 0.934 to 0.942; thus, the parameter can be confidently used to characterize the adsorption system. The derived Temkin constant related to the heat of adsorption (B) is consistent with this finding, in which the values lie between 0.0048 and 0.0065 kcal mol^-1^ that implies the occurrence of physical sorption (physisorption).

The adsorption of MB by N_*g*_-CuGA is dependent on temperature. It was found that the adsorption capacity decreased as temperature increased, indicating an exothermic behavior^55^; this behavior was also consistent with the physisorption dominant of the system. Furthermore, the decrease of the adsorption affinity at higher temperatures was well-represented by the parameters of the Langmuir model. Specifically, the monolayer adsorption capacity, *Q*_L_, was found to decrease at high adsorption temperature. Subsequently, the *K*_L_ value, which shows the adsorption affinity, was also reduced by increasing temperature. At higher temperature, the solute molecules tend to have high kinetic energy, which increases the randomness of the molecules. Consequently, the solute and adsorbent surface interactions are delayed due to the high mobility of the solute molecules^56^.

The three-parameter isotherm models (i.e., Sips and Redlich–Peterson), which incorporate the Langmuir and Freundlich models, were often utilized to validate the two-parameter models. The fitting results suggest that both Sips and Redlich–Peterson could well-describe the experimental data based upon the R^2^ values. Nonetheless, it can be seen in Fig. 5e that the fitting lines of Redlich–Peterson tend to overestimate the experimental data before the plateau region and underestimate after the plateau. Therefore, it can be affirmed that the Sips model would best describe the adsorption isotherm. Another point to be noticed is that the *Q*_exp_ values for all investigated temperatures have closest resemblance with the *Q*_S_ from the Sips model. Meanwhile, the *Q*_L_ of the Langmuir model and *Q*_RP_ (*K*_RP_/*a*_RP_) of the Redlich–Peterson model yield a considerably lower value than the *Q*_exp_. Thus, it can be stated that the Sips model can satisfactorily describe the adsorption isotherm. The heterogeneity of the adsorption system can be predicted from the *s*_p_ and *a*_s_ parameters of the Sips model. The *s*_p_ values are closer to 1, indicating that the adsorbent tends to possess homogeneous adsorption sites. Furthermore, the *a*_s_ values were far from zero, indicating that the Sips model would reduce to Langmuir rather than Freundlich^57^. It is worth mentioning that the adsorption capacity of N_*g*_-CuGA toward MB is high at the neutral pH and room temperature (i.e., 30 °C), denoting the prospect of this adsorbent material for low-cost and practical adsorption application.

#### Adsorption thermodynamics

The thermodynamic parameters of the adsorption of MB by N_*g*_-CuGA are given in Table 3. A negative Δ*G* and a positive Δ*S* indicate that the adsorption process proceeds spontaneously. The positive value of Δ*S* can be related to the release of water molecules on the surface of the adsorbent as the adsorbate molecules are attached^58^. A negative Δ*H* suggests the exothermic behavior of the adsorption system, which is in accordance with the prediction by the Temkin model.

**Table 3:** Thermodynamics parameters of MB adsorption onto N_*g*_-CuGA.

Parameter	Temperature (K)		
	303	313	323
Δ*G* (kJ mol^-1^)	− 37.70	− 37.98	− 39.49
Δ*H* (kJ mol^-1^)	− 10.45		
Δ*S* (J mol^-1^ K^-1^)	89.30		

#### Adsorption kinetics

**Figure 6:**
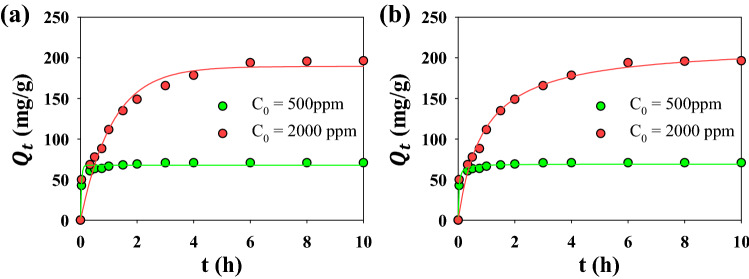
Adsorption kinetics of MB onto N_*g*_-CuGA. The solid lines represent data fitting to the Pseudo first order (**a**) and Pseudo second order (**b**); symbols represent experimental data.

Adsorption kinetics can demonstrate the adsorption rate, which is a crucial factor to describe the efficiency of the process. Pseudo first order and Pseudo second order were used to model the adsorption kinetic data and determine MB adsorption rate into N_*g*_-CuGA. The fitting of the models was shown by the solid line passing through the adsorption data points in Fig. 6, and the fitting parameters were presented in Table 4. The Pseudo second order model was well-converged with the data, which is indicated by higher *R*^2^ (i.e., 0.988 and 0.958) than the Pseudo first order (i.e., 0.972 and 0.941).

**Table 4:** Experimental maximum capacity and calculated isotherm parameters for the adsorption of MB onto N_*g*_-CuGA.

Model	Initial concentration of MB (mg/L)	
	500	2000
*Q*_exp_ (mg g^-1^)	70.745 ± 0.077	196.399 ± 0.114
**Pseudo first order**		
*Q*_1_ (mg g^-1^)	67.732 ± 1.043	189.367 ± 8.069
*k*_1_ (h^-1^)	29.837 ± 4.217	0.894 ± 0.124
R^2^	0.972	0.941
**Pseudo second order**		
*Q*_2_ (mg g^-1^)	69.189 ± 0.750	216.436 ± 10.386
*k*_2_ (g mg^-1^ h^-1^)	0.627 ± 0.091	0.005 ± 0.001
R^2^	0.988	0.958

The initial MB concentration (*C*_0_) was shown to affect the adsorption rate and the number of adsorbed MB molecules (*Q*_t_). As shown in Fig. 6, the MB adsorption increases dramatically within 4 h adsorption for the system with *C*_0_ = 2000 ppm. Meanwhile, the adsorption proceeded faster at a lower *C*_0_ of 500 ppm; wherein, the equilibrium was reached only after 2 h. This is in accordance with the estimated adsorption rate value by Pseudo second order (*k*_2_), where *k*_2_ was found to decrease at higher *C*_0_. At a higher *C*_0_ of 2000 ppm, the high number of adsorbate molecules rapidly fill the vacant adsorption sites during the beginning; and therefore, the affinity of the adsorbent with the remaining adsorbate molecules in the bulk solutions become weaker, and the adsorption rate was slowing down^59^. The adsorption capacity was also dependent on the *C*_0_, and it was found that the adsorption capacity (*Q*_exp_) increases at higher *C*_0_; which is in good accordance with the adsorption capacity predicted by Pseudo second order (*Q*_2_). At higher *C*_0_, the abundance of adsorbate molecules provides a driving force to suppress the adsorbate-adsorbent mass transfer resistance. Thus, more adsorbate molecules can be adsorbed^60–62^.

#### Effect of adsorbent dose and salinity

**Figure 7:**
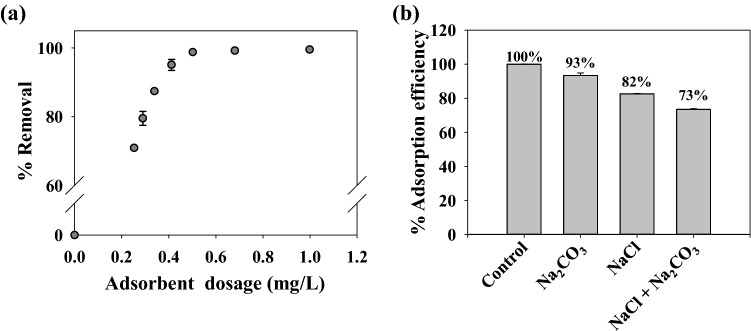
(**a**) Effect of adsorbent dosage on %removal of MB, at *C*_0_ = 70 ppm. (**b**) Effect of salinity on the adsorption of MB onto N_*g*_-CuGA at a different salt concentration (Na_2_CO_3_ = 20 ppm, NaCl = 300 ppm, and their combination).

The adsorbent dose is an essential factor in designing an economic adsorption process. The effect of adsorbent dose was studied at an initial MB concentration (*C*_0_) of 70 ppm, mimicking dye concentration in textile industry wastewater^63^. At a low adsorbent dosing, saturation in the adsorption sites of adsorbent may occur before the adsorption equilibrium is achieved. In contrast, an excessive adsorbent dosing may generate unnecessary vacant adsorption sites since the adsorbate had been completely removed before reaching its maximum adsorption capacity^64^. Figure 7a shows the variation of MB %removal at different adsorbent doses. The highest %removal of 99.9 was obtained at an adsorbent dose of 0.5 mg/L and the usage of adsorbent at higher dosing (> 0.5 mg/L) may leads to inefficient adsorbent usage^65^.

Some salts such as NaCl and Na_2_CO_3_ were commonly found in textile wastewater at a concentration of 300 ppm and 20 ppm, respectively^63^. Thus, it is necessary to elucidate their effect on the %removal of MB by N_*g*_-CuGA. Figure 7b shows that the reduction of adsorption efficiency in the presence of salt ions. The reduction can be related to the attachment of salt cations on the surface of the adsorbent, which consequently decreases the MB attraction to the surface of N_*g*_-CuGA. It was also worth mentioning that the adsorption efficiency further deteriorated at the higher salt concentration. At a given Na_2_CO_3_ concentration of 20 ppm, the repulsion effect causes a 7% decrease of the %removal. Meanwhile, 18% and 27% decrease on the %removal was occurred at higher salt concentration, i.e., NaCl = 300 ppm and mixture of NaCl/Na_2_CO_3_ = 300/20ppm.

For comparison purposes, the reported adsorption capacity of MB on different metal-linker coordination adsorbents is listed in Table 4. Compared with the CuGA MOF^22^, the adsorption capacity of MB by N_*g*_-CuGA was 53.09% higher, indicating the synergistic effect of the N-functional groups from Gly addition. Furthermore, the higher adsorption capacity of N_*g*_-CuGA compared to CuGA can be attributed to the immense pore volume and pore diameter, i.e., 0.56 cc/g and 23.25 nm for N_*g*_-CuGA and 0.43 cc/g, and 8.6 nm for CuGA. The bigger pores of N_*g*_-CuGA may facilitate the insertion of the MB molecules into its matrix, thus increasing the amount of MB molecules that can be trapped. A similar occurrence was found on the amine-functionalization of MOF-Fe, in which the amine-MOF-Fe was shown to possess a higher adsorption capacity and a bigger mean pore diameter (18.63 nm) than the unfunctionalized one (3.26 nm)^66^. The presence of the electron lone pairs of the –NH_2_ groups on the N_*g*_-CuGA may account for the better attraction of positive-charged MB^66^, thus improving its adsorptivity. It is also worth mentioning that the N_*g*_-CuGA has a higher MB adsorption capacity than other materials listed in Table 5, demonstrating the potential usage of N_*g*_-CuGA as a highly adsorptive adsorbent for MB removal.

**Table 5:** MB adsorption capacity on various metal-linker coordination adsorbents.

Name	Metal ion	Linker	Additive	Qe (mg/g)	Temp (°C)	pH	Ref
N_*g*_-CuGA	Cu	GA	Gly	190.81	30	6	This work
CuGA MOF	Cu	GA	–	124.64	30	6	^22^
MOF-Fe	Fe	H_2_BDC	–	149.25	NA	9	^66^
Cu-BTC	Cu	BTC	–	47.77	25	7	^67^
UiO-66	Zr	H_2_BDC	–	90.48	25	9	^68^
Reduced graphene oxide	–	–	–	61.5	30	–	^49^
	–	–	Gly	98.9	30	–	

#### Reusability

**Figure 8:**
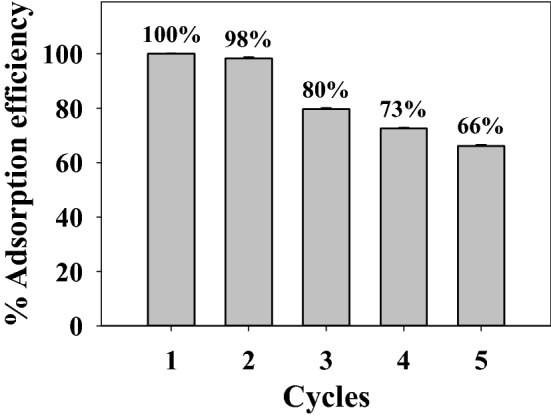
The adsorption efficiency of N_*g*_-CuGA for the MB removal after five adsorption–desorption cycles.

The ability of N_*g*_-CuGA adsorbent to undergo several repeating adsorption cycles was investigated by performing a 5-cycles reusability study. Figure 8 shows that N_*g*_-CuGA can maintain high adsorption efficiency of 98% up to the second cycle. The adsorption capacity was then declined significantly at the third to fifth cycle which might be due to the occurrence MB remained in the adsorption sites of N_*g*_-CuGA.

## Conclusion

Nitrogen-grafted CuGA (N_*g*_-CuGA) was successfully synthesized by adapting the metal–ligand complexation principle. The synthesis was done by mixing an equimolar amount of Cu, GA, and Gly at pH 8 under ambient temperature. The grafting was confirmed by the occurrence of Gly characteristic peak in the FTIR spectra of N_*g*_-CuGA. The nitrogen functional group of Gly shown to synergistically promote the adsorption capacity of N_*g*_-CuGA by 1.53-fold higher compared to its non-grafted analogous CuGA. The adsorptivity of N_*g*_-CuGA shown to be closely related to the pH, temperature, and the salinity of the system. N_*g*_-CuGA was able to maintain 80% of adsorption efficiency up to the third adsorption cycle.
